# Tissue Resources for the Functional Annotation of Animal Genomes

**DOI:** 10.3389/fgene.2021.666265

**Published:** 2021-06-21

**Authors:** Michèle Tixier-Boichard, Stéphane Fabre, Sophie Dhorne-Pollet, Adeline Goubil, Hervé Acloque, Silvia Vincent-Naulleau, Pablo Ross, Ying Wang, Ganrea Chanthavixay, Hans Cheng, Catherine Ernst, Vicki Leesburg, Elisabetta Giuffra, Huaijun Zhou, Collaborative Working Group, Catherine Taragnat

**Affiliations:** ^1^University Paris-Saclay, INRAE, AgroParisTech, GABI, Jouy-en-Josas, France; ^2^GenPhySe, INRAE, ENVT, ENSAT, Castanet-Tolosan, France; ^3^University Paris-Saclay, CEA, IRCM, Fontenay-aux-Roses, France; ^4^UC Davis Department of Animal Science, University of California, Davis, Davis, CA, United States; ^5^USDA-ARS Avian Disease and Oncology Laboratory, East Lansing, MI, United States; ^6^Department of Animal Science, Michigan State University, East Lansing, MI, United States; ^7^USDA-ARS, Fort Keogh Livestock and Range Research Laboratory, Miles City, MT, United States

**Keywords:** tissue sampling, repository, mammals, bird, cryopreservation, genome

## Abstract

In order to generate an atlas of the functional elements driving genome expression in domestic animals, the Functional Annotation of Animal Genome (FAANG) strategy was to sample many tissues from a few animals of different species, sexes, ages, and production stages. This article presents the collection of tissue samples for four species produced by two pilot projects, at INRAE (National Research Institute for Agriculture, Food and Environment) and the University of California, Davis. There were three mammals (cattle, goat, and pig) and one bird (chicken). It describes the metadata characterizing these reference sets (1) for animals with origin and selection history, physiological status, and environmental conditions; (2) for samples with collection site and tissue/cell processing; (3) for quality control; and (4) for storage and further distribution. Three sets are identified: set 1 comprises tissues for which collection can be standardized and for which representative aliquots can be easily distributed (liver, spleen, lung, heart, fat depot, skin, muscle, and peripheral blood mononuclear cells); set 2 comprises tissues requiring special protocols because of their cellular heterogeneity (brain, digestive tract, secretory organs, gonads and gametes, reproductive tract, immune tissues, cartilage); set 3 comprises specific cell preparations (immune cells, tracheal epithelial cells). Dedicated sampling protocols were established and uploaded in https://data.faang.org/protocol/samples. Specificities between mammals and chicken are described when relevant. A total of 73 different tissues or tissue sections were collected, and 21 are common to the four species. Having a common set of tissues will facilitate the transfer of knowledge within and between species and will contribute to decrease animal experimentation. Combining data on the same samples will facilitate data integration. Quality control was performed on some tissues with RNA extraction and RNA quality control. More than 5,000 samples have been stored with unique identifiers, and more than 4,000 were uploaded onto the Biosamples database, provided that standard ontologies were available to describe the sample. Many tissues have already been used to implement FAANG assays, with published results. All samples are available without restriction for further assays. The requesting procedure is described. Members of FAANG are encouraged to apply a range of molecular assays to characterize the functional status of collected samples and share their results, in line with the FAIR (Findable, Accessible, Interoperable, and Reusable) data principles.

## Introduction

A coordinated genome-wide identification of functional elements in multiple species represents an invaluable resource for the dissection of genotype-to-phenotype relationships. The Functional Annotation of Animal Genome (FAANG) initiative (Andersson et al., 2015; Giuffra et al., 2019) supports the international community in the production of comprehensive maps of functional elements in the genomes of domesticated animal species. An early aspiration of the FAANG Consortium was to create a framework for organizing data standardization, collection, and sharing from many groups (Tuggle et al., 2016). The FAANG data portal^1^ has been established to ensure high-quality and rich supporting metadata to describe its farmed and companion animals, samples, and related data sets (Harrison et al., 2018).

In order to generate an atlas of the functional elements driving genome expression in different biological conditions, the FAANG strategy has been to sample many tissues from different species, sexes, ages, and production stages. A consensus was reached at a workshop convened at Plant and Animal Genome (2014) as reported by Andersson et al. (2015). Two FAANG pilot projects [FRAGENCODE for INRAE (Institut national de la recherche agronomique), France, and FarmENCODE for University of California, Davis (UCD), United States] were initially funded to support this effort. This article details the sampling and storage procedures and describes the metadata collection of the reference samples collections for four livestock species (cattle, pig, chicken, goat) realized by these two pilot projects, as well as the guidelines for their possible future use.

## Animals

### Species and Population of Origin

A prerequisite was to sample species with high-quality genome assemblies. Then, taxonomic diversity was considered: mammals and birds have been sampled, and among mammals, ruminants and non-ruminants have been selected. This article describes the sampling done for *Bos taurus*, *Capra hircus*, *Sus scrofa*, and *Gallus gallus* by INRAE and UCD.

A large choice of breeds is available within each species worldwide. Well-characterized breeds were prioritized and selected for sampling. Regarding cattle, Holstein breed is the most widely used dairy cattle as is Hereford for beef cattle. Regarding goat, one of the two mostly used dairy breeds (the Alpine) was sampled, to allow for comparisons between two species of ruminants for milking traits. Regarding pigs, the sampling included Large White as a dam line and Yorkshire as a sire line. Regarding chickens, the White Leghorn breed was chosen as it provides the genetic basis for numerous experimental lines and is widely used for white egg production. A control line from a selection experiment was sampled, as well as an F1 crossbred obtained from two highly inbred White Leghorn lines differing in disease resistance.

### Selection History of the Animal

Animals were chosen so as to be representative of their breed in order to be used as a reference for future studies. They all had a known pedigree, and some of them could also have produced progeny. If possible, frozen semen was collected from males to be able to produce progeny in the future.

Both sexes were sampled, two males and two females for each genetic type (Table 1). Adult animals were sampled for all species, considering they were in a stable period for gene expression. They already had performance records, obtained in known environmental conditions. A limited number of physiological states were recommended in order to have more assays from the same tissue in the same individual and have replicates across laboratories and to maximize comparisons across species. For Alpine goats and Large White pigs, blood cells were also sampled at different ages in the young males, in order to allow for a longitudinal analysis of immune traits in the same individual. These young males were the progeny of the adult females that were slaughtered or were closely related. For Large White pigs, blood cells were sampled monthly from weaning at 1 month of age until slaughter at 8 months of age. For Alpine goats, only two blood samples could be collected in the young males.

**TABLE 1:** Number of animals sampled according to species and sex, showing age at sampling according to sex, and reproductive stage for females.

Species	Breed	Female	Age (days)	Stage	Male	Age (days)	Castrated male	Age
Bos taurus	Holstein	2	1250, 1532	Lactating	2	539, 602	–	–
Bos taurus	Hereford (Line 1)	2	420	Post-ovulatory	2	420	–	
Capra hircus	Alpine	2	1697, 2072	Pregnant and lactating	2	Days 49 to 246	–	
Sus scrofa	Large White	2	592, 595	lactating	2	Days 25 to 255		
Sus scrofa	Yorkshire						2	170
Gallus gallus	White Leghorn	2	387	Laying	2	387		
Gallus gallus	White Leghorn F1 crossbred (Lines 6x7)	2	140	Sexually mature but not yet in lay	2	140		

### Flock/Herd/Owner

Animals sampled by INRAE came from its experimental facilities, except for the two Holstein bulls with registration numbers FR2832014033 and FR4934530986 that were purchased from a breeding center in France (*Origen plus*). There is no legal uncertainty regarding the ownership of the biological material sampled from experimental animals, neither for the bulls and their semen, sold by *Origen plus* for research use, without any further conditions.

For the UCD project, two bulls with registration number of 43497294 and 43496857 and two heifers with registration numbers of 43497060 and 43496864 were raised for 12 months in animal facility at Fort Keogh Livestock and Range Research Laboratory in the US Department of Agriculture–Agricultural Research Service (USDA-ARS) and were then transferred to Animal Facility at UCD for another 2 months before the samples collection. Two male and two female chickens were raised at the USDA, ARS, Avian Disease and Oncology Laboratory (ADOL). Two littermate male pigs were provided by the Michigan State University Swine Teaching and Research Center in East Lansing, MI.

### Environmental Conditions

#### Production System

Cattle: Holstein cows were provided by the INRAE facility Le Pin (latitude 48°44′6.6′ North; longitude 0°9′58.8″ East). They were raised with a mixed system: in closed barns with freedom of movement from November to April, on grassland from May to October. Inside the barn, they were fed *ad libitum* with a “winter” diet composed of 48% maize silage, 24% green silage with dehydrated pulp, 21% concentrate, 7% rapeseed meal, and 150 g minerals. On grassland, they received daily 2 kg of concentrate with additional complementation with maize silage if necessary. Hereford bulls and heifers were raised at the Fort Keogh Livestock and Range Research Laboratory of USDA-ARS (latitude 45°47′15.4896″ North, longitude 108°29′21.4944″ West) with the same mixed system. Inside the barn, they were fed *ad libitum* with a bull’s ration of 20% corn, 10% hay, 5% supplement, and 65% silage and a Heifer ration of 39.5% hay, 3.5% supplement, and 57% silage.

Goats: Alpine goats and bucks were provided by the INRAE facility located in Bourges (latitude 47°1′59.98″ North; longitude 2°39′0″ East). They were raised in closed barns. Females were fed *ad libitum* with dry hay composed of *Dactylis* and alfalfa. Lactating females received in addition 1.2 kg of concentrate (19% total proteins, 5.3% lipids, 26% starch, 9% raw cellulose, 1% calcium) per day. Males were fed *ad libitum* with dry hay from grass and received 0.6 kg of the same concentrate.

Pigs: Large White pigs were provided by the INRAE facility located in Rouillé (latitude 46°25′0.02″ North; longitude 0°3′0″ East). They were housed in groups on straw and fed twice a day with a complete diet (13.5% total proteins, 3% lipids, 6.8% raw cellulose, and 6% ashes, supplemented with minerals, vitamins, and amino acids lysine and methionine) with a total amount of 2.7 to 3.2 kg/day for females, according to pregnancy stage, and of 2.7 to 3.5 kg/day for males according to body weight. Water was provided *ad libitum*. Boars were isolated at the time of semen collection. Two littermate castrated male Yorkshire pigs were provided by the Michigan State University Swine Teaching and Research Center in East Lansing, MI (latitude 42°44′15.5472″ North; longitude 84°29″1.6368″ West). Following weaning at 21 days of age, pigs were housed in groups of 10 with other castrated male pigs, on rounded metal slat flooring with fiberglass-gated sides. Pigs were moved to a grow-finish pen at 65 days of age in groups of 14 pigs, with metal-gated sides and a fully slatted concrete floor. Pigs were fed *ad libitum* with a commercial diet meeting or exceeding the National Research Council (2012) nutritional requirements for each stage of development. Feed was delivered using one self-feeder per pen with 0.61 m of linear feeder space in the nursery and 0.77 m of linear feeder space in the grow-finish pen. Water was provided *ad libitum* with a single nipple drinker in each pen.

Chickens: White Leghorn chickens were provided by the INRAE experimental unit facility located in Nouzilly (latitude 47°32′38″ North; longitude 0°44′41″ East). Adults were kept in individual cages for pedigree control and egg recording. Females received 16 h of light per day in a single cycle, and males received 10 h of light per day. Ambient temperature was set at 20°C for females and 19°C for males. They were fed *ad libitum* with a complete diet containing either 17.5% total proteins (supplemented with methionine, lysine, cysteine), 3.3% lipids, 2.6% cellulose, 40% starch, 13% ashes, and 4% calcium for females, or 12.5% total proteins (supplemented with lysine and methionine), 2.8% lipids, 4.2% cellulose, 4.4% ashes, and 0.75% calcium for males (detailed list of compounds can be provided upon request). The Line 6 × 7 F1 chickens were provided by the USDA, ARS, ADOL (latitude 42°44′15.5472″ North; longitude 84°29′1.6368″ West) located in East Lansing, MI. Male adults were housed in Horsfall-Bauer isolation units that received 8 h of light per day and kept at 21–27°C. They were fed *ad libitum* “starter” feed crumbles.

#### Vaccination Program

Cattle: cows were vaccinated against pulmonary infections and enteric diseases during the rearing phase, and each year thereafter for enteric diseases, at the start of the winter period (Rispoval^®^ RS-BVD) and at the end of it (Coglavax^®^). In addition, they were vaccinated against neonatal diseases (Trivacton^®^ 6) in the last month of pregnancy.

Female goats were vaccinated against blue tongue virus, and a serological test was performed to check for the absence of brucellosis. As kids, they received only a treatment against coccidiosis (Vecoxan^®^ or equivalent).

Pigs were not vaccinated, but serological tests were performed to check absence of brucellosis, parvovirus, Aujeszky virus, and porcine reproductive and respiratory syndrome.

Chickens received a complete vaccination program from hatch to the adult stage, with vaccinations against Marek disease, Newcastle disease, Gumboro disease, infectious bronchitis, rhinotracheitis, infectious anemia, encephalomyelitis, coccidiosis, and egg drop syndrome (detailed list of vaccines with the calendar can be provided upon request).

### Physiological Status at Sampling

Date of birth was recorded for each animal so that age at sampling was precisely known (Table 1). Reproductive stage was determined for females; early pregnancy stage was detected in goats upon sampling the uterus (Table 1). Animals were fasted for at least 12 h before slaughter.

## Tissue Collection

For INRAE, collection took place after slaughter that was realized according to the authorized practices, without chemical anesthesia. As tissue sampling after death is not submitted to an official permit, the ethical approval was needed in case of blood sampling on live animals. For mammals, blood samples were collected in the context of the approvals (APAFIS/project#): 334-2015031615255004_v4 and 333-2015031613482601_v4 (pigs), 3066- 201511301610897_v2 (cattle), 03936.02, and 8613-2017012013585646_v4 (goats). Chicken immune cells were obtained from spleen sampled after slaughter (no need for animal experiment authorization). For UCD, tissues were collected following Protocol for Animal Care and Use #18464, approved by the Institutional Animal Care and Use Committee (IACUC), UCD. Collection protocols are available from https://data.faang.org/protocol/samples, and a link to each standard protocol is included along with collection description.

For male Large White pigs, scalding was not performed as slaughter took place in an experimental facility at INRAE. Scalding was performed for Yorkshire pigs that were slaughtered in a commercial slaughterhouse. Scalding may expose testis to high temperature stress, but these pigs were castrated, so that no effect of scalding was encountered.

Small cubes of 0.5 × 0.5 × 0.5 cm^3^ were sampled from all solid tissues at INRAE (INRA_SOP_tissue_sampling_ 1a_20160721.pdf) with a total of 2 to 20 replicates from each tissue. At UCD, cross sections of tissue were minced/homogenized using scalpel/scissors to collect subsamples (UCD_SOP_50_TissueCollection_20160520.pdf). In addition, for some complex tissues or special cell preparations, specific protocols were developed: they are mentioned in *Tissue collection* and listed in Supplementary Table 1.

The aim of tissue collection was to cover a wide range of tissues for a comprehensive approach of genome annotation. Because of anatomical differences between species, particularly between mammals and birds, some tissues were not collected in all species. Parallel sampling by experts was performed to minimize time to sample preservation; the order of sampling was recorded because it provided an estimate of time elapsed since death. It may be estimated that sampling time varied from 30 min for one chicken to 2 h for one cow. Tissues known to be more susceptible to degradation, such as pancreas or brain, were sampled first. Thus, the majority of tissues were sampled within 30 min postmortem; it was shorter than 30 min for pancreas (<10 min) and brain in mammals, as well as for all tissues in chickens. It was within an hour for digestive and reproductive tracts for cattle, sheep, and pig, except for Holstein where it was up to 2 h.

To classify tissues into different sets, the following parameters were considered including functional importance, standardization of sampling, realized assays, and specific cell preparations. As a consequence, several sets of tissues have been identified (Table 2).

**TABLE 2:** List of tissues or tissue sections of a given organ, with number of aliquots in the collection, according to species and functional tract.

Tissue	Tissue set	*Bos taurus*	*Capra hircus*	*Sus scrofa*	*Gallus gallus*	Total	Functional Tract
Abdominal fat	1	59	44	48	44	195	Adipose tissue
Subcutaneous fat	1	44	44	44		132	Adipose tissue
Heart	1	64	40	44	40	188	Cardio-respiratory tract
Lung	1	56	41	44	20	161	Cardio-respiratory tract
Trachea	2	8		4	8	20	Cardio-respiratory tract
Abomasum	2	24				24	Digestive system
Cecum	2	24			24	48	Digestive system
Colon	2	68	44	46		158	Digestive system
Duodenum	2	64	40	44	20	168	Digestive system
Esophagus	2	8		4	8	20	Digestive system
Gall bladder	2	8				8	Digestive system
Gizzard	2				39	39	Digestive system
Ileum	2	64	40	44	20	168	Digestive system
Jejunum	2	64	40	44	20	168	Digestive system
Liver	1	88	79	76	60	303	Digestive system
Omasum	2	8				8	Digestive system
Reticulum	2	8				8	Digestive system
Rumen	2	8				8	Digestive system
Salivary gland	2	8				8	Digestive system
Stomach	2			4		4	Digestive system
Pigment epithelium ey	2	8				8	Epithelium
Skin	1	96	48	44	40	228	Epithelium
Tongue superficial	2	8				8	Epithelium
Trachea epithelium	3	44	20	40		104	Epithelium
Bone marrow	2	52	43	47	16	158	Immune tissue
Lymph nodes	2	32	14	16		62	Immune tissue
Peyer’s patches	2	30	44	44		118	Immune tissue
Spleen	1	56	56	52	24	188	Immune tissue
Thymus	2	30	21	34		85	Immune tissue
Cerebellum	2	49	41	44	12	146	Nervous system
Frontal lobe (cortex)	2	68	44	48	16	176	Nervous system
Hypothalamus	2	16	8	12	12	48	Nervous system
Medulla	2	8				8	Nervous system
Olfactory lobe	2	32	20	44		96	Nervous system
Pons	2	8				8	Nervous system
Spinal cord	2	8				8	Nervous system
Thalamus	2	8				8	Nervous system
Cervical lining	2	4				4	Female reproductive system
Fornix vagina	2	4				4	Female reproductive system
Infundibulum oviduct	2	8			10	18	Female reproductive system
Isthmus fallopian tube/hen isthmus	2	13			10	23	Female reproductive system
Corpus luteum	2	22	19	2		43	Female reproductive system
Ovarian cortex	2	26	20	19	4	69	Female reproductive system
Ovarian follicle	2	4			10	14	Female reproductive system
Oviduct	2	16	22	22		60	Female reproductive system
Oviductal ampulla/hen magnum	2	18			10	28	Female reproductive system
Theca ovarian follicles	2				10	10	Female reproductive system
Uterus/shell gland	2	32	22	22	10	86	Female reproductive system
Uterovaginal gland	2				10		Female reproductive system
Prostate	2	4				4	Male reproductive system
Bulbourethral	2	4				4	Male reproductive system
Epididymis	2	34	10	10		54	Male reproductive system
Seminal vesicle	2	26	22	22		70	Male reproductive system
Spermatozoon	2	20	10	10	72	112	Male reproductive system
Testis	2	26	40	40	22	128	Male reproductive system
Vas deferens	2	4			7	11	Male reproductive system
Adrenal cortex	2	52	20	44	6	122	Secretory tissue
Pituitary	2	13	8	6	4	31	Secretory tissue
Mammary	2	24	29	20		73	Secretory tissue
Pancreas	2	52	44	48	24	168	Secretory tissue
Thyroid	2	16	7	9		32	Secretory tissue
Cartilage	2	52	44	47	27	170	Skeleton
Tongue muscle	2	8				8	Striated muscle
Diaphragm	2	8				8	Striated muscle
Dorsal muscle	1	40	40	40		120	Striated muscle
Pectoral muscle	1				32	32	Striated muscle
Sartorius muscle	1				36	36	Striated muscle
Bladder	2	8		2		10	Urinary tract
Kidney	2	16		4	16	36	Urinary tract
Renal cortex	2	40	36	40		116	Urinary tract
Renal medulla	2	44	40	44		128	Urinary tract
Ureter	2	4				4	Urinary tract
Urethra	2	8				8	Urinary tract
Grand total	1878	1204	1322	743		5137	

### Set 1: The Standard Set

This set corresponds to the tissues for which collection is easy to standardize and which will be studied with several assays. It included liver, spleen, lung, heart, skin, fat depot, muscle, and peripheral blood mononuclear cells (PBMCs, i.e., lymphocytes).

Liver samples were taken from the edge of the organ, avoiding proximity with gallbladder and avoiding blood vessels. Gallbladder was collected in the Hereford cow.

The entire spleen was extracted from the abdominal cavity for mammals. Capsule part was removed, and cubes of 0.5-cm-long edges were isolated from the bands. For birds, spleen tissue was either processed the same (UCD project), or a specific procedure was implemented to separate spleen cells from red blood cells, in order to avoid contamination of immune cells by platelet cells (INRA_SOP_chicken_splenocytes_sampling_20160721.pdf).

Lung samples were taken from the edge of the organ, avoiding large bronchioles. Left and right lobes were separately collected in the Hereford cow.

Heart muscle was collected for all animals, with separate collection of left and right ventricles and atria at UCD.

In mammals, a large piece of skin (15 × 10 cm) from the groin of the right leg was extracted from the carcass. This location was chosen to limit the presence of hairs or bristles. Nevertheless, the entire piece of skin was shaved with a scalpel and finally rinsed with phosphate-buffered saline (PBS) 1 × solution to remove hairs. First, using a circular skin biopsy punch tool (8 mm in diameter) and thereafter a scissor to separate epidermis and dermis from subcutaneous fat tissue, individual skin biopsies were isolated and frozen. When the coat color exhibited different types of pigmentation, biopsies were sampled from contrasted areas, either white or black areas in the Holstein and pale or sustained brown color in the Alpine goat. In chickens, sections of 0.5-cm diameter were sampled from an unfeathered area on the internal face of the leg.

Subcutaneous fat and perirenal abdominal fat were collected for mammals. In addition, mesenteric adipose that lies with layers of the peritoneal mesothelium connecting the small and large intestine was collected at UCD. In chickens, abdominal fat was collected around the gizzard (INRAE) or in the abdominal cavity (UCD).

Muscle samples were taken from the longissimus lumborum for all mammals and from two different muscles for chickens: pectoralis major (“white fibers”) from the breast and sartorius or semimembranosus (“red fibers”) from the leg. A section from the center of each muscle was collected to avoid adipose tissue and major connective tissue structures. In addition, biceps femoris (bottom/outside round), gluteus medius (top sirloin), longissimus dorsi (ribeye/loin), and psoas major (tenderloin) were also collected in the Hereford cow.

Blood was sampled using EDTA as anticlotting agent. For mammals, whole blood was used to prepare peripheral lymphocytes (called hereafter PBMCs). In order to get a sufficient number of each type of cells, sampling was repeated from the jugular vein between two and five times according to age and species, in the weeks preceding slaughter. Blood was immediately handled to separate PBMCs, as described in FAANG protocols for cattle and goats (INRA_SOP_PBMC_purification_cattle_caprine_20160504.pdf), and for pigs (INRA_SOP_PBMC_seperation_swine_blood_20160504.pdf). As it was not possible to immediately perform cell sorting after each sampling, all PBMCs were frozen before sorting, in order to standardize the preparation of defined populations of lymphocytes.

### Set 2: Tissues Requiring Specific Sampling Protocols

#### Brain Tissues

These tissues are extremely sensitive to degradation. A specific team needs to be in charge of sampling them in the shortest delay after death. In mammals, the brain was separated in four regions: cerebellum, frontal lobe, olfactory lobe, and hypothalamus (INRA_SOP_cattlebrain_sampling_20171104.pdf). The pituitary gland was sampled, and its posterior/anterior parts were separated for Holstein only. In Hereford, the cortex was separated in three subregions: frontal, parietal, and temporal. In addition, pigment epithelium eye, spinal cord, medulla, pons, and thalamus were collected too. In Yorkshire pigs, only cerebellum, cortex, and hypothalamus were collected. In chickens, olfactory bulbs were not dissected, and three parts were dissected: cerebellum, frontal lobe, and hypothalamus. Pituitary was also sampled at INRAE.

#### Digestive Tract

##### Mammals

In the Hereford cow and the Yorkshire pig, parotid salivary glands were collected and minced/homogenized using scalpel/scissors. Tongue muscle was collected from approximately halfway in the organ. Superficial tongue sample was collected from the papillary epithelium using a scalpel to separate it from the muscle.

The whole digestive tract was set on a table. The different gut sections were identified, and 10- to 15-cm portions of each region were isolated between two ligatures, after pushing the maximal amount of the content on each side. Then, each portion was open and rinsed with PBS before sampling. Reticulum, rumen, and abomasum were collected at UCD. At INRAE, duodenum, jejunum, ileum, and colon were collected, keeping the mucosa and the muscular layer together before transfer into individual cryotubes, whereas at UCD, mucosa was scraped from the lumen of the tissue using a clean glass slide. Tissue remaining after mucosal scrapping was saved as smooth muscle sample. This conditioning was applied to abomasum, duodenum, jejunum, ileum, cecum, and colon for which three parts (whole, mucosa, and smooth muscle) were collected. The caudal mesenteric node was identified as the most distal lymph node of the mesenteric chain, and square sections of 0.5 × 1 cm were sampled.

##### Chicken

Caeca were sampled in place of colon, and gizzard was taken in addition to gut sections. Portions were isolated in a similar way as the one used in mammals, and sections were rinsed with PBS before transferring to individual cryotubes. In addition, mucosal scrapping was performed for half of the aliquots sampled for duodenum, jejunum, and ileum.

#### Secretory Organs: Mammary Gland, Pancreas, Kidney, Adrenal Glands, and Thyroid

A specific protocol was set up for mammary gland to sample the secretory parenchyma (INRA_SOP_mammarygland_sampling_20171104.pdf). All females were lactating, and the mammary gland was sampled for the cow, goat, and sow.

The entire kidney organs were extracted from the carcass. Capsule was first removed. Each kidney was separated by the middle in two pieces to observe differently colored cortex and medulla parts. Bands of 0.5 cm large were cut with scalpel through the cortex part, and cubes with 0.5-cm-long edges were isolated from the bands and frozen. Cubes of 0.5-cm-long edges were individually dissected from the medulla apparent pyramids. Ureter, bladder, and urethra were also collected in the Hereford cow.

As chicken kidney does not have a similar cortex/medulla structure, it was sampled as a homogenous tissue with several aliquots.

Pancreas and thyroid were collected as quickly as possible without being further dissected. Thyroid could not be found in some individuals.

The cortex of the adrenal glands, which produces cortisol and aldosterone, was dissected in mammals, whereas the whole gland was sampled in chickens.

#### Reproductive Tract

##### Mammals

Ovaries underwent a specific dissection protocol, which allowed separating ovocytes from granulosa cells (INRA_SOP_oocytes-granulosa_mammals_sampling_20160721.pdf). In addition, small cubes of 0.5-cm side were cut out from the ovarian cortex and the luteal body (corpus luteum), rinsed in PBS, and transferred individually to cryotubes. There was no luteal body available for sampling in sows.

Uterus samples were taken in the main body of the uterus and included both the mucosa and the muscular layer for all species. Further dissection was implemented for the Hereford cow: tissues from caruncular and intercaruncular regions were collected separately; endometrium was collected by scraping the inner layer of the uterus, and myometrium was isolated from serosa and endometrium by scraping and scalpel. Fourteen sections were then separated: ampula (contralateral to corpus luteum), ampula (ipsilateral to c*orpus luteum*), infundibulum (contralateral to *corpus luteum*), infundibulum (ipsilateral to corpus luteum), isthmus of fallopian tube (contralateral), isthmus of fallopian tube (ipsilateral), uterine myometrium, ovarian section (without corpus luteum), uterine endometrium – caruncular (contralateral to corpus luteum), uterine endometrium – caruncular (ipsilateral to corpus luteum), uterine endometrium – intercaruncular (contralateral to corpus luteum), uterine endometrium – intercaruncular (ipsilateral to corpus luteum), Fornix vagina, and cervical lining.

Male reproductive tissues were dissected to separate testis from epididymis and seminal vesicle. Testis was sampled as small cubes or slices, and seminiferous tubules were also dissected and pretreated to implement Hi-C protocol (see *Specific preparations*). For boars and bucks, semen was obtained from epididymis after slaughter and was conditioned with a tris, citrate, and glucose solution supplemented with 15% (vol/vol) egg yolk and 5% (vol/vol) glycerol as described in Pini et al. (2018). For bulls, semen was collected in an accredited artificial insemination center, which provided semen straws that were transferred into a liquid nitrogen tank in order to preserve their fertilizing ability for future functional studies and/or production of progeny.

At UCD, three sections of the epididymis were further separated (caput, corpus, and tail), and prostate, vas deferens, and bulbourethral gland were collected in cow.

##### Chicken

In females, theca and granulosa cells were separately dissected from the largest follicles, ordered by decreasing size. In addition, very small follicles were preserved in five aliquots.

The presence of an egg in the shell gland was recorded. Several sections of the reproductive tract were sampled: infundibulum (also called oviductal ampula, the closest section from the ovary, where fecundation takes place), magnum (where the albumen is produced), the isthmus, and shell gland (equivalent to the uterus). In addition, glands located at the uterovaginal junction (between the shell gland and the cloaca) were also sampled, as they play a key role in the preservation of spermatozoa after insemination.

Six weeks before slaughter, adult males were trained for 2 weeks in order to collect semen by massage twice a week during 2 weeks. Semen volume, motility, and viability were recorded for each ejaculate. Semen was then diluted with a cryoprotectant agent and frozen in 0.5-mL straws, which were identified by a color code and the unique animal number (INRA_SOP_freezinggallussemen_20200401.pdf). Straws were stored in liquid nitrogen to preserve fertilizing ability of spermatozoon.

At slaughter, as testes are internal organs of homogenous structure, they were separated from vas deferens: small cubes (0.5 cm^3^) were cut out either from testis or from vas deferens, rinsed in PBS and transferred individually to cryotubes. There are no seminal vesicles in chickens.

#### Immune Tissues: Thymus, Bone Marrow, and Lymph Nodes

In mammals, three types of lymph node were sampled: the caudal mesenteric node, the node located at the trachea-bronchial bifurcation, and the neck lymph nodes. Thymus was collected. Yellow bone marrow cubes of 0.5-cm-long edges were individually collected in the hemimedullary cavity of the tibia. Peyer patches were sampled in cattle, except for Holstein females, in pigs, and in goats.

In chickens, there are no lymph nodes, as well as no Peyer patch. For this species, immune cells were separated from the spleen, as described in *Set 1: the standard set*. Thymus and bone marrow were collected.

#### Bone and Cartilage

In mammals, the tibia bone of the anterior right leg was extracted from the carcass and cut by the middle in two pieces with a butcher knife. In chicken, the whole tibia bone was cut in 10 small pieces, which were stored in individual cryotubes.

Cartilage was sampled from the femur in mammal species, as described in INRA_SOP_cartilage-sampling_20171117.pdf.

### Set 3: Specific Cell Preparations

#### Immune Cells

CD4^+^ and CD8^+^ cells were sorted from PBMCs previously prepared for mammals, following protocols adapted to each species (INRA_SOP_sorting_cattle_CD_cells_20171201.pdf; INRA_SOP_sorting_caprine_CD_cells_20171201.pdf; INRA_SOP_sorting_swine_CD_cells_20160504.pdf). For chickens, CD4^+^ and CD8β^+^ cells were sorted from purified spleen cells, as described in INRA_SOP_sorting_chicken_CD_cells_20180213.pdf. Alveolar macrophages were separated according to a specific protocol that was applied to mammals only (INRA_SOP_alveolar-macrophages_mammals_sampling_20160721.pdf).

These cells have been stored in liquid nitrogen for future studies.

#### Epithelial Cells

Tracheal epithelium was dissected from the trachea for mammals only, (INRA_SOP_tracheal_epithelium_mammals_sampling_20160721.pdf) and then stored at −80°C, as other tissues.

## Tissue Conditioning

### Standard Procedure

Aliquots for homogenous tissues were stored without any buffer into individual cryotubes and immediately snap frozen into liquid nitrogen (INRA_SOP_tissue_sampling_1a_20160721.pdf) on the collection site and then transported in dry ice and placed at −80°C for long-term conservation. Individual aliquots were placed in a single tube, and supernumerary aliquots for a given tissue were pooled, as described in INRA_SOP_tissue_ aliquots_sampling_1b_20160721.pdf.

### Handling Cellular Heterogeneity Within a Tissue

For very heterogeneous tissues, it is obvious that individual aliquots would not be comparable. Thus, aliquots were not stored individually but were pooled for future studies; this was the case for hypothalamus, pituitary, lymph nodes, spleen, thyroid, and thymus, as described in INRA_SOP_ tissue_aliquots_sampling_1b_20160721.pdf.

An additional option was to save tissue morphology for further cellular dissection. For that aim, we used the Optimal Cutting Temperature (OCT) compound to perform embedding of a slice of tissue of 1 cm long, 0.5 cm wide, and 0.3 cm thick in a mold placed on dry ice, (INRA_SOP_ tissue_sampling_protocol_6_20180426.pdf). This was done on one aliquot for most tissues, in order to make possible future analysis on identified cell types using either histology or laser microdissection. It was then stored at −80°C.

### Specific Preparations

Two types of specific analyses were planned for FAANG: Hi-C and ATAC-Seq. Dedicated cell preparation was performed on fresh samples at the site of sampling in view of HiC, as described in INRA_SOP_liver_spleen_mammarygland_ forHiC_sampling_20160721.pdf and INRA_SOP_testis_forHiC_ sampling_20160721.pdf, or in view of ATAC-seq, as described in INRA_SOP_ATAC-seq_AG_v1_20160805.pdf for liver, spleen, and CD4^+^/CD8^+^ cells from pigs, goats, cattle, and chickens.

At present, ATAC-Seq analyses are known to be possible from snap-frozen tissues. Thus, we can consider that most tissues from this collection are now available for ATAC-seq analyses.

Altogether, 17 specific sampling or conditioning protocols and four general sampling protocols can be found in https://data.faang.org/protocol/samples.

## Use of the Samples

### Sample Description

**FIGURE 1:**
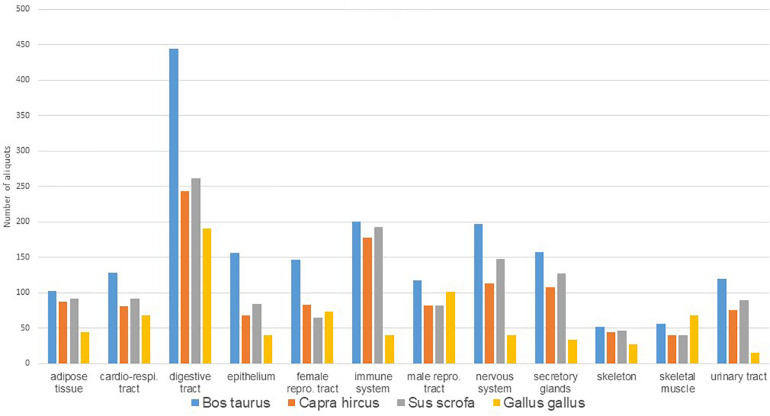
Distribution of number of aliquots available in Biosamples according to species and main tissue types.

A total of 3,949 tissue aliquots from the FRAGENCODE project are currently identified with a Biosamples ID (1,184 for cattle, 1,148 for goats, 1,188 for pigs, and 429 for chickens; Supplementary Table 2), and a total of 462 samples, with two or three aliquots each, from the FarmENCODE project are available with Biosamples ID (SAMEA4454482-4455404 for chickens, SAMEA4454615-4455481 for cattle, and SAMEA4454570-4454614 for pigs; Supplementary Table 3). Ontologies such as UBERON or BRENDA have been used to describe the samples. Additional tissues are stored at INRAE, which require additional curation to get a final ontology, particularly for chicken female reproductive tract, as well as for pigmented or non-pigmented skin in all species. The total number of aliquots preserved is currently 5,137, representing 73 different tissues or tissue section, which can be grouped into 12 main functional categories (Table 2). The number of aliquots is shown in Figure 1 according to species and functional category. There are 21 tissues collected for all four species, and 37 collected for the three mammals. Cattle showed the highest number (67) of tissues or tissue sections collected. Within a species and a sex, the number of tissues was the same per individual. Females had more subsections of the reproductive tract sampled than males, so that the total number of samples was higher for females. Chickens had a lower number of tissues sampled because of anatomical differences (i.e., no lymph nodes, no mammary gland, no subcutaneous fat in the White Leghorns) and also had a lower number of aliquots because of the smaller size of tissues, particularly for brain, but also for kidney, where cortex and medulla are not distinguished as in mammals.

There is no ontology commonly used for breed name and raising conditions; this remains to be validated and used at the international level. A list of breeds can be obtained from the Food and Agriculture Organization database^2^, but the naming of breeds is not necessarily harmonized across countries. Consequently, this information has been described with some details in *Animals* of this article, which also provides the link to the sampling protocols.

### Quality Control

To validate sampling protocols and verify the RNA integrity, RNA extraction was performed from an aliquot of different tissues or cell types for liver, muscle, mammary gland, lung, spleen, heart, and immune cells. Samples were homogenized in TRIzol Reagent (Life Technologies, Carlsbad, CA, United States) using an Ultra Turrax (IKA) set at 26,000 revolution/min. Total RNAs were extracted according to the manufacturer’s instructions (Life Technologies) using optional instructions. Insoluble materials were removed after homogenization by centrifugation at 12,000 *g* for 10 min at 4°C before adding chloroform.

RNA yield and purity were monitored by spectrophotometry (NanoDrop ND-1000). RNA integrity was assessed using an Agilent (Santa Clara, CA, United States) 2100 Bioanalyzer and RNA 6000 nano kits. RNA quality was evaluated using the RNA integrity number (RIN) value introduced by Agilent (Schroeder et al., 2006). RIN values for liver RNA ranged from 7.8 to 8.8 for mammals and from 8.8 to 9.1 in chicken. For immune cells, higher RIN values were obtained in mammals (from 7.7 to 9.7, with a majority of samples with an RIN > 9) than in chickens (from 5.1 to 8.1), which could be due to the more complex separation procedure from spleen cells.

### Transcriptome Studies

The FRAGENCODE project aimed at improving the genomic annotation of four species (cattle, goat, chicken, and pig). This was achieved by performing molecular assays on tissue dissociated cells (liver) and on sorted primary cells (CD4^+^ and CD8^+^ T lymphocytes) from two males and two females of each species. These assays included RNA-seq, ATAC-seq, and Hi-C to characterize the transcriptome, the chromatin accessibility, and the genome 3D topology in these cells, respectively (Foissac et al., 2019). Additional work was carried out using these RNA-seq datasets for the annotation of long-non-coding RNAs (lnRNAs) (Jehl et al., 2020).

The collection is being used to complete the reference transcriptome of six tissues (cerebellum, lung, kidney, dorsal skin, skeletal muscle, small intestine/Ileum), in addition to the liver datasets reported by Jehl et al. (2020). This additional annotation is being conducted in the frame of the H2020 FAANG project GENE-SWitCH^3^.

The FarmENCODE project was initiated to functionally annotate farm animal genomes (chicken, pig, and cattle), particularly in the regulatory elements. The eight distinct tissues (adipose, cerebellum, cortex, hypothalamus, liver, lung, skeletal muscle, and spleen) from two males of each species have been used to identify tissue-specific expressed mRNAs and lnRNAs across three species (Kern et al., 2018). The ATAC-seq assay on these eight tissues from pig and cattle was performed to analyze chromatin accessibility conservation across mammals (Halstead et al., 2020). Furthermore, ChIP-seq (four histone modification marks and CTCF) assays across three species and DNase-seq in chickens were performed to annotate dynamic chromatin states across tissues and species (Kern et al., 2021).

## Procedure to Request Samples

All tissue aliquots are available for any researcher, provided that the work planned is scientifically sound or will be useful to improve a methodology for FAANG, for example.

Because sanitary conditions have been recorded, health regulations should not be a limitation to access to samples. Since 2014, an EU regulation makes it compulsory to comply with the Nagoya protocol of the Convention on Biological Diversity, regarding access and benefit sharing from the use of genetic resources. In France, there is no access measure for genetic resources from domestic animals, so there is no limitation to access to these samples. United States is not party to the Nagoya protocol, and there is no condition for access either.

The only request for a user of an FAANG sample described in this article is to acknowledge the origin of samples by referring to the present article and to share the results obtained with all partners of the FAANG initiative. A moderate cost can be requested to cover preservation and shipment costs, in order to keep the tissue collection available in the long term.

For the INRAE collection, the procedure to request tissue samples is to create an account on the CRB-Anim web portal, https://crb-anim.fr/access-to-collection/#. The portal provides access to the 3,949 tissue samples also declared in Biosamples. Discovering the whole FRAGENCODE sample collections is possible by a simple browse that will provide information about species, breed, and sample type. You need to identify yourself by creating an account in order to get more precise information about the tissue type and to request samples of interest to you with the advanced search procedure. For any specific question, a contact address is available (contact-crb-anim@inrae.fr).

Access to the UCD collection is possible by contacting the corresponding author from UCD and will be made available from the CRB-Anim web portal in the course of 2021.

## Conclusion

The FAANG tissue collection set up by INRAE and UCD illustrates the concept of biobank for research in genomics of domestic animals. Whereas the Biosamples database sets the reference identification for biological samples that can be used for research and makes possible to connect molecular data with these samples, additional procedures set up by a biobank are needed to manage the conservation and distribution of samples to the scientific community. To facilitate sample sharing, documenting sampling protocols as well as animal physiological status and raising conditions is needed and has been described in this protocol article. Combining different methods or types of analyses on a limited set of reference animals avoids the random noise due to variation among experiments and makes proposing a reference data set for genome structure and function possible. Furthermore, cryoconservation of spermatozoa enables the production of progeny from these males, for which gene expression profile will have been studied. Once a reference set is defined, targeted experiments with additional sampling will be able to identify deviations from the reference, as long as sampling protocols as well as animal physiological status and raising conditions are known. It is thus highly recommended to upload all sampling protocols in https://data.faang.org/protocol/samples. The preservation and distribution of reference samples, as well as of samples from well-defined experiments, are expected to decrease the number of animals included in future experiments. At present, biobanking stem cells is becoming the priority in order to facilitate the production of organoids, also an alternative to *in vivo* experiments.

## Data Availability Statement

Publicly available datasets were analyzed in this study. This data can be found here: crb-anim.fr/access-to-collection/# for FRAGENCODE project. In addition, excel files extracted from Biosamples for both FRAGENCODE and FarmENCODE projects are provided in Supplementary Data, Files 2, 3.

## Ethics Statement

The animal study was reviewed and approved by the INRA Val de Loire Ethical Committee for animal experimentation for blood sampling of live animals and received the approvals (APAFIS/project#): 334-2015031615255004_v4 and 333-2015031613482601_v4 (pigs), 3066- 201511301610897_v2 (cattle), 03936.02 and 8613-2017012013585646_v4 (goats). No approval is needed in France for tissue sampling after slaughter. For UCD, tissues were collected following Protocol for Animal Care and Use #18464, approved by the Institutional Animal Care and Use Committee (IACUC), University of California, Davis.

## Collaborative Working Group

Catherine Taragnat, Cecile Berri, Déborah Jardet, Estelle Godet, Fabrice Laurent, Gilles Gomot, Hughes Dardente, Isabelle Grasseau, Jean-Philippe Dubois, Joel Gautron, Nadine Gérard, Pascale Quéré, Roger-Paul Lavocat, Rozenn Dalbies-Tran, Sonia Métayer, Sylvain Marthey, Vincent Coustham, and Xavier Druart.

## Author Contributions

MT-B, SF, SD-P, AG, HA, SV-N, and EGi designed and implemented the FRAGENCODE project. PR, YW, GC, HC, CE, VL, and HZ designed and implemented the FarmENCODE project. MT-B drafted the manuscript with HZ and EGi. Authors from the collaborative working group CB, VC, RD-T, HD, XD, J-PD, JG, EGo, GG, NG, IG, FL, SMé, PQ, and CT organized the sampling and brought a special expertise on a tissue or a particular species. DJ, R-PL, and SMa identified all samples and handled the data from collection to submission to Biosamples for FRAGENCODE project. All authors contributed to the article and approved the submitted version.

## Conflict of Interest

The authors declare that the research was conducted in the absence of any commercial or financial relationships that could be construed as a potential conflict of interest.

## Footnotes

1 https://data.faang.org/home

2 http://www.fao.org/dad-is/en/

3 https://www.gene-switch.eu/
